# Sex-Specific Differences in MicroRNA Expression During Human Fetal Lung Development

**DOI:** 10.3389/fgene.2022.762834

**Published:** 2022-04-11

**Authors:** Nancy W. Lin, Cuining Liu, Ivana V. Yang, Lisa A. Maier, Dawn L. DeMeo, Cheyret Wood, Shuyu Ye, Margaret H. Cruse, Vong L. Smith, Carrie A. Vyhlidal, Katerina Kechris, Sunita Sharma

**Affiliations:** ^1^ Division of Environmental and Occupational Health, National Jewish Health, Denver, CO, United States; ^2^ Division of Pulmonary Sciences and Critical Care Medicine, Department of Medicine, University of Colorado School of Medicine, Aurora, CO, United States; ^3^ Department of Biostatistics and Informatics, Colorado School of Public Health, University of Colorado-Denver Anschutz Medical Campus, Aurora, CO, United States; ^4^ Division of Bioinformatics and Personalized Medicine, Department of Medicine, University of Colorado School of Medicine, Aurora, CO, United States; ^5^ Environmental and Occupational Health, Colorado School of Public Health, Aurora, CO, United States; ^6^ Channing Division of Network Medicine, Division of Pulmonary and Critical Care Medicine, Department of Medicine, Brigham and Women’s Hospital, Boston, MA, United States; ^7^ Children’s Mercy Hospital and Clinics, Kansas City, MO, United States

**Keywords:** microRNA, lung development, pulmonary disease, sex-specific, gene expression, human

## Abstract

**Background:** Sex-specific differences in fetal lung maturation have been well described; however, little is known about the sex-specific differences in microRNA (miRNA) expression during human fetal lung development. Interestingly, many adult chronic lung diseases also demonstrate sex-specific differences in prevalence. The developmental origins of health and disease hypothesis suggests that these sex-specific differences in fetal lung development may influence disease susceptibility later in life. In this study, we performed miRNA sequencing on human fetal lung tissue samples to investigate differential expression of miRNAs between males and females in the pseudoglandular stage of lung development. We hypothesized that differences in miRNA expression are present between sexes in early human lung development and may contribute to the sex-specific differences seen in pulmonary diseases later in life.

**Methods:** RNA was isolated from human fetal lung tissue samples for miRNA sequencing. The count of each miRNA was modeled by sex using negative binomial regression models in DESeq2, adjusting for post-conception age, age^2^, smoke exposure, batch, and RUV factors. We tested for differential expression of miRNAs by sex, and for the presence of sex-by-age interactions to determine if miRNA expression levels by age were distinct between males and females.

**Results:** miRNA expression profiles were generated on 298 samples (166 males and 132 females). Of the 809 miRNAs expressed in human fetal lung tissue during the pseudoglandular stage of lung development, we identified 93 autosomal miRNAs that were significantly differentially expressed by sex and 129 miRNAs with a sex-specific pattern of miRNA expression across the course of the pseudoglandular period.

**Conclusion:** Our study demonstrates differential expression of numerous autosomal miRNAs between the male and female developing human lung. Additionally, the expression of some miRNAs are modified by age across the pseudoglandular stage in a sex-specific way. Some of these differences in miRNA expression may impact susceptibility to pulmonary disease later in life. Our results suggest that sex-specific miRNA expression during human lung development may be a potential mechanism to explain sex-specific differences in lung development and may impact subsequent disease susceptibility.

## Introduction

Sex-specific differences in human lung maturation begin *in utero*. Using histologic staging, the fetal lungs of human females are more mature than males from 20 to 32 weeks of gestation (Naeye et al., 1974). Measurements of the lecithin-sphingomyelin ratio in fetuses between 28 and 40 weeks of gestation also suggest that human female lungs are about 1.2–2.5 weeks more mature than male lungs (Torday et al., 1981). However, the time point at which sexual differences emerge in human lung development, the biological pathways differing by sex, and the regulatory mechanisms underlying these differences are not well understood. Using transcriptomic profiling in human fetal lung tissue, we have previously shown that sexual dimorphism in human lung development occurs as early as the pseudglandular stage (Kho et al., 2016a). In part, these differences are hypothesized to be driven by sex hormones and sex-specific differences in endogenous corticosteroid handling (Boucher et al., 2014). For example, estrogens are known to alter surfactant production (Seaborn et al., 2010). In addition, glucocorticoids, which are known to be essential to lung development, are influenced by endogenous androgens during development in a sex-specific manner (Provost et al., 2013). While these mechanisms are known to impact the structural development of the lung in the later stages of gestation, the impact of sex-specific differences in early lung development have not been fully elucidated. Thus, better characterization of sex-specific differences in early lung development, a critical time period of airway growth and branching morphogenesis, may offer insight into the biological mechanisms that underlie sex-based differences in respiratory disease.

Epigenetic regulation of human development is well recognized and may provide insights into additional mechanisms that explain sex-specific differences in lung maturation. Furthermore, an understanding of the sex-specific epigenetic changes that regulate human lung development may also help to understand the developmental origins of diseases that exhibit sex-specific differences in prevalence. For example, the ability to characterize sex-specific differences in epigenetic processes may offer insight into the biological mechanisms that enhance susceptibility to childhood respiratory diseases among males, including respiratory distress syndrome, bronchopulmonary dysplasia, and childhood asthma (Naeye et al., 1971).

Of particular interest are microRNAs (miRNAs), small RNAs (∼21–24 nucleotides) that are important regulators of gene expression. miRNAs target protein-coding genes by cleavage of target messenger RNA (mRNA), inhibition of translation, and/or mRNA deadenylation (Bhaskaran et al., 2009). A single miRNA can have many gene targets, with 30% of the human genome thought to be miRNA targets (Lewis et al., 2005). As such, miRNAs are essential regulators of many critical biological processes. Animal models have previously demonstrated the importance of miRNAs in lung development, including airway growth and branching morphogenesis. Proteins that are essential for miRNA processing appear to have a prominent role in murine lung development. For example, a seminal study showed that the inactivation of Dicer, a protein required to generate mature miRNAs, leads to significant branching defects in mouse embryonic lung tissue resulting in large epithelial pouches (Harris et al., 2006). In addition, Ago1 and Ago2, members of the Argonaute family which regulate small RNAs, were shown to be enriched in the distal epithelium and mesenchyme of the early developing lung, suggesting miRNA mediated gene regulation occurs in a localized manner (Lü et al., 2005). Specific miRNAs have also been shown to have a significant impact on the developing lung, including the overexpression of miR-127 in early fetal rat lung development, which causes defective branching and terminal bud formation (Bhaskaran et al., 2009).

Animal models have also established that there are sex-specific differences in miRNA expression within the developing lung (Mujahid et al., 2013) and that these sex-specific differences may impact subsequent disease risk. Given this existing body of evidence, it is plausible that there are sex differences in miRNA levels during early human lung development, and that these sex-specific differences in miRNA expression may impact subsequent disease risk. However, to-date sex-specific differences in miRNA expression have not been explored in human biospecimens, particularly using high-throughput profiling methods. To address this existing knowledge gap, we generated the first miRNA sequencing profiles of early human fetal lung tissue samples to investigate differential miRNA expression between developing male and female lungs during the pseudoglandular stage of lung development. We hypothesize that differences in miRNA expression are present in early human fetal lung development between sexes, and this may be a mechanism to explain sex-specific differences in susceptibility to pulmonary diseases later in life.

## Methods

### Sample Acquisition and Phenotypic Characteristics

Human fetal lung tissue samples were collected as part of a prenatal tissue retrieval program sponsored by the National Institute of National Child Health and Development, the University of Maryland Brain and Tissue Bank for Developmental Disorders (Baltimore, MD), and the Center for Birth Defects Research (University of Washington; Seattle, WA). The study was designated an institutional review board (IRB) exempt protocol by the University of Missouri-Kansas City Pediatric IRB, Partners Human Research Committee IRB, and the Colorado Multiple Institutional Review Board (COMIRB). Due to sample de-identification, limited maternal and fetal phenotypic characteristics were available for each sample including gestational age and sex. Sample sex was previously confirmed based on paired gene expression data (Kho et al., 2016a) by classifying samples as female or male based on their expression of X- and Y-chromosome genes. The age of samples was determined using estimated days post-conception. Intrauterine cigarette smoke exposure (based on placental cotinine concentration) (Vyhlidal et al., 2013) is a well-known confounder of fetal lung development and was directly measured in the samples using the Cotinine Direct ELISA kit (Calbiotech, Spring Valley, CA). Unmeasured confounders were accounted for using the RUV method described below in miRNA profiling.

### miRNA Profiling

We extracted total RNA from 30 mg of homogenized prenatal lung tissue with the miRNeasy Mini Kit per manufacturer instructions (Qiagen; Valencia, CA, United States). Samples were block-randomized by age, sex, and smoke exposure status to four batches of miRNA library preparation (Small RNA Sequencing Kit v3 for Illumina Platforms; Bio Scientific) and sequencing (HiSeq2500; Illumina; San Diego, CA, United States). The resulting reads were trimmed for low-quality base calls and Illumina adaptor sequence using cutadapt (Martin, 2011), and then mapped to counts of known miRNAs using miR-MaGiC (Russell et al., 2018) with reference to the miRbase (Kozomara and Griffiths-Jones, 2014) v22.1 database. Samples passing quality-control (≥1 × 10^5^ mapped reads, having available phenotypic information, and being within the 7–17 weeks pseudoglandular period) and autosomal miRNAs passing pre-filtering criteria (non-zero counts in at least 25% of samples; passes DESeq2 independent filtering algorithm (Bourgon et al., 2010); and not mapped to X or Y chromosome) were tested for differences by sex. Using RUV-Seq (Risso et al., 2014), we also obtained four RUVr factors representing unmeasured miRNA expression heterogeneity for inclusion in regression models.

### Statistical Modeling of Sex Differences

We evaluated if miRNA features differed by sex in two respects. First, we identified autosomal miRNAs with varying average expression levels between male and female samples regardless of sample estimated post-conception age (i.e., effect present across the entire pseudoglandular developmental stage). Using DESeq2 (Love et al., 2014), we modeled the count of each miRNA (outcome) by sample sex (explanatory variable of interest; indicator variable for male or female), adjusting for age and age squared (age^2^) (Torday et al., 1981) (age is measured in days post-conception; age^2^ is a quadratic term to capture potential non-linear effects), smoke exposure (≥or <7.5 ng cotinine/g placenta), technical batch (indicator variable for batch 1, 2, 3, or 4), and four inferred covariates to capture additional unmeasured confounders (k = 4 RUVr components). A statistically significant difference in mean miRNA levels by sex was defined by a likelihood ratio test at a multiple testing corrected *q*-value (Storey, 2002) < 0.05.

Second, we screened for autosomal miRNAs with sex-specific “age-trajectories” using a likelihood ratio test to evaluate whether adding an age-by-sex interaction significantly improved model fit. A significant interaction (*q*-value < 0.05) implies that the pattern of miRNA expression levels by age were distinct between male and female participants. For example, a miRNA may increase in male samples, yet decrease or remain the same in female samples.

Functional Interpretation of Sex-Differing miRNAs. To interpret the functional impact of miRNAs differentially expressed by sex and with sex-specific age trajectories, we used miRNAtap (Pajak and Simpson, 2021) to identify predicted gene expression targets regulated by each miRNA. In addition, we sorted the list of miRNAs tested by descending *p*-value and used pre-ranked gene set enrichment analyses in miEAA to conduct pathway analyses (Subramanian et al., 2005; Kern et al., 2020). Statistically significant enrichment in a pathway (*q*-value < 0.05) indicates that miRNAs associated with the pathway appear at the top of the list (lower *p*-values) more frequently than would be expected by random chance. Annotations of miRNAs to pathways were based on the miRWalk 2.0 database, as curated by the miEAA developers (Dweep et al., 2011).

### Study Reproducibility

Additional methodological details are available online (Supplementary File S1: Detailed Methods). Code for the statistical analyses and processed miRNA-sequencing data is available at github. com/chooliu/miRNASexDimorphismFetalLung. Raw and processed miRNA-sequencing data are pending deposition approval at the NCBI GEO database.

## Results

### Characteristics of the Human Fetal Lung Tissue Samples

**FIGURE 1:**
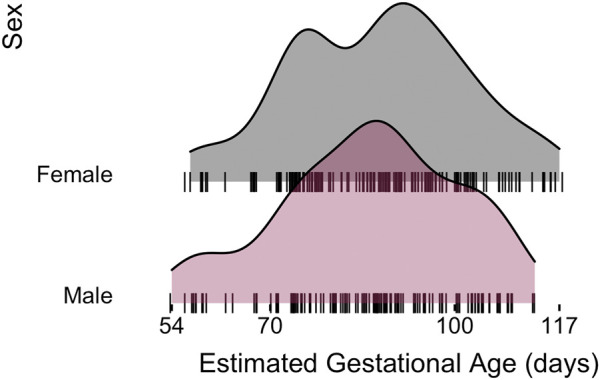
Human fetal lung tissue samples used for this analysis demonstrates a similar distribution of estimated gestational ages observed in each sex.

miRNA-sequencing profiles were generated on human fetal lung tissue samples ranging in gestational age from 54 to 117 days within the pseudoglandular histological stage of human lung development. The final set of 298 samples was comprised of 166 male (56%) and 132 (44%) female samples (Table 1). The same approximate distribution of age ranges was observed in each sex (Figure 1; no significant difference in distribution based on Wilcoxon signed-rank test, *p* = 0.41). Based on placental cotinine, our previously validated biomarker of intrauterine smoke exposure, 139 (47%) of the samples had been exposed to intrauterine smoke.

**TABLE 1:** Characteristics of human fetal lung samples analyzed.

	Female	Male	All
N	132	166	298
Age (dpc)	87.0 (76.0, 96.5)	89.0 (76.0, 96.0)	87.0 (76.0, 96.0)
IUS (exposed)	62 (47.0%)	77 (46.4%)	139 (46.6%)

### Lung miRNA Expression Profiles During the Pseudgolandular Stage of Human Lung Development

After pre-processing and quality control, our final dataset consisted of 809 miRNAs measured in 298 human lung samples. Of the known sample physical characteristic variables, gestational age appeared to explain the largest variability in miRNA expression, with age explaining anywhere from 0 to 52% of the variance in the expression of each miRNA. In comparison, sex tended to explain only up to 8% of each miRNA’s variance.

### Differential Expression of miRNAs by Sex

**FIGURE 2:**
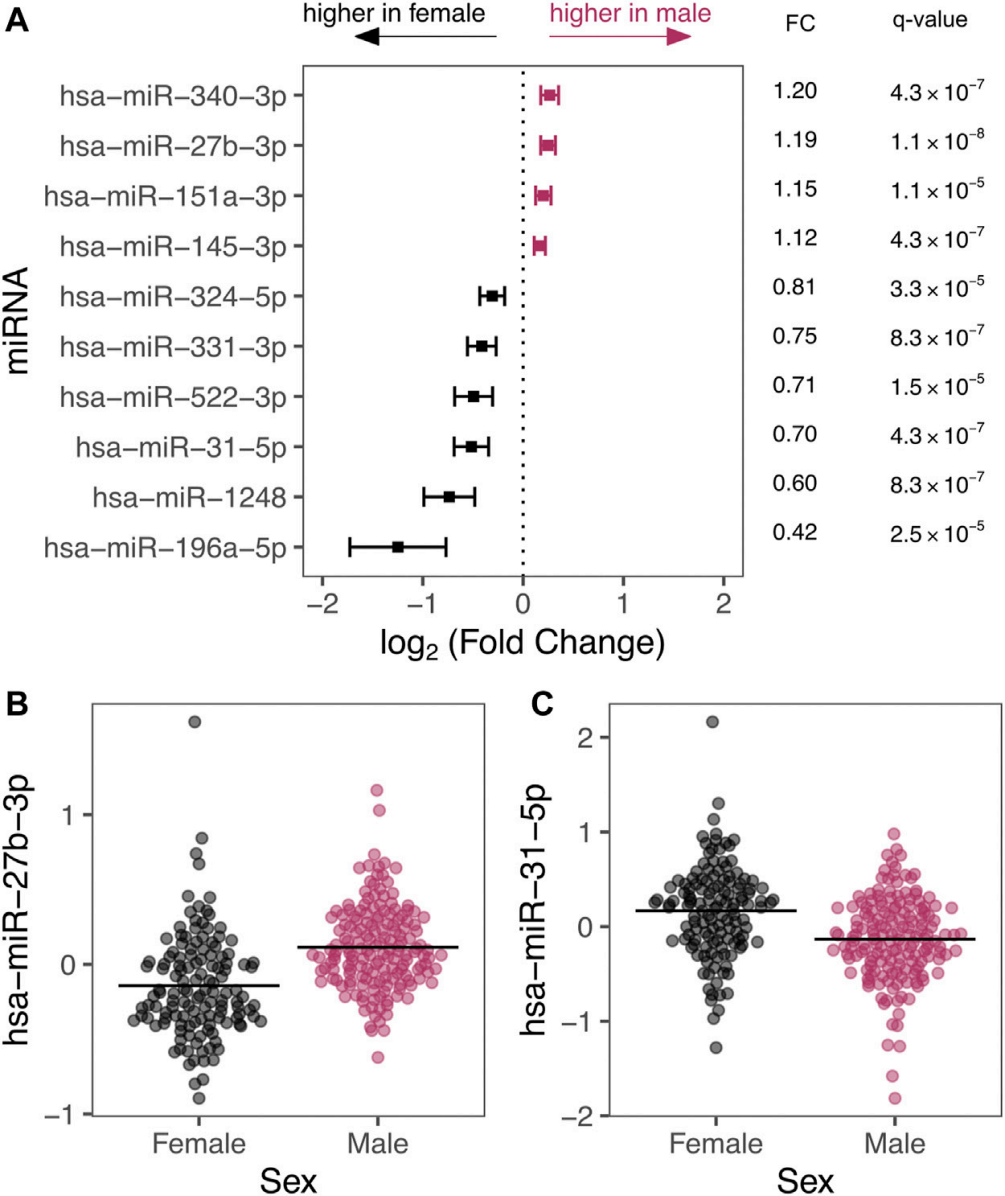
**(A)** Top 10 miRNAs by *p*-value are depicted with their effect size expressed as log_2_ (fold change) with 95% confidence intervals. Estimated fold changes (miRNA counts in males compared to female samples) and q-values are also presented. **(B,C)** show example scatter plots of residualized miRNA levels (*y*-axis) plotted by sex (*x*-axis). Residuals are displayed to illustrate sex-differences after adjusting for covariates including, age, age (Torday et al., 1981), smoke exposure, technical batch, and the four RUV-inferred covariates to emphasize effects of sex.

We detected 93 autosomal miRNAs with significant differential expression by sex after correcting for covariates. A subset of differentially expressed miRNAs are shown in Figure 2 (full list in Supplementary Table S1). The predicted mRNA targets for each of these miRNAs are also provided in Supplementary Table S1. Interestingly, these miRNAs differentially expressed by sex include miRNAs predicted to regulate gene expression targets with known roles in pseudoglandular lung development: For example, *hsa-miR-27b-3p* is predicted to regulate peroxisome proliferator-activated receptor-*γ* (PPAR*γ*) and *hsa-miR-196a-5p* is predicted to regulate multiple Hox transcription factors including homeobox B7 (HOXB7).

Pathway enrichment analyses also support the possibility that sex-specific miRNAs may collectively regulate biological pathways relevant to pseudoglandular lung development. Sex-associated miRNAs were significantly enriched in numerous pathways (gene set enrichment *q*-value < 0.05; full results in Supplementary Table S2), including pathways with previously identified roles in lung development, such as Wnt-signaling, vascular endothelial growth factor (VEGF), fibroblast growth factor (FGF), and transforming growth factor beta (TGF*β*) signaling pathways. Sex-associated miRNAs were also significantly enriched with genes associated with androgen and estrogen signaling.

### Sex-Specific miRNA Expression Trajectories During Human Lung Development

**FIGURE 3:**
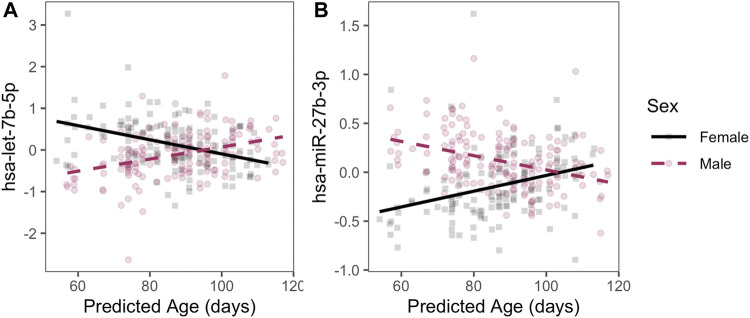
Examples of sex-specific trajectories. Two miRNAs with statistically significant age-by-sex interactions are plotted with residualized expression (*y*-axis) across age (*x*-axis) during the pseudoglandular period differs by sex (indicated by color). Residuals are displayed to illustrate sex-by-age differences after adjusting for smoke exposure, technical batch, and the four RUV-inferred covariates to emphasize patterns by age and sex.

In addition, we detected 129 autosomal miRNAs with significant linear age-sex interactions; that is, a sex-specific age trajectory of miRNA expression over the course of the pseudoglandular period. Two examples of miRNAs with a significant age-sex interaction—*hsa-let-7b-5p* and *hsa-miR-27b-3p*—have their expression levels by age plotted for illustration (Figure 3; *q*-value < 0.05, full list of significant results in Supplementary Table S3). Sex-associated miRNAs identified in this manner also were significantly enriched in multiple pathways, including epidermal growth factor (EGF) and muscarinic acetylcholine receptor (mAChR) signaling (full results in Supplementary Table S4).

## Discussion

While sex-specific differences that exist during *in utero* lung development are well-recognized by histological measurements, much remains to be understood about the differences between males and females during early lung development and their impact on subsequent disease risk. Our study applies high-throughput, untargeted miRNA-sequencing to rare human fetal lung tissue samples and characterizes differences in miRNA expression by sex for the first time, highlighting novel regulatory features and biological pathways that differ by sex in early prenatal lung development. Sex-specific differences in these miRNAs may potentially explain differences in lung maturation and in subsequent respiratory disease susceptibility later in life that differs between males and females.

We detected 93 miRNAs with significantly different average expression levels by sex during the pseudoglandular stage of human lung development, as well as 129 miRNAs with significant age-by-sex interactions indicative of sex-specific miRNA expression age-trajectories across the pseudoglandular stage. The sex-specific miRNAs include some miRNAs previously shown to impact lung development. For example, *hsa-let-7b-5p* was both identified as significantly differentially expressed (higher levels in males on average throughout pseudoglandular period) and identified as a miRNA with a sex-specific trajectory (expression increasing by age in males, but decreasing by age in females). The *let-7* family of miRNAs are well-known regulators of human lung cell proliferation (Johnson et al., 2007), and in mouse models appear to play crucial roles in regulating both the timing and the cell differentiation processes associated with airway branching, possibly mediated by let-7’s downstream impacts on TGF*β* signaling and epithelial-mesenchymal interactions (Gulman et al., 2019).

We also identified other miRNAs with differences by sex that have not been as richly characterized but are predicted to regulate gene expression targets essential to lung development. For example, *hsa-miR-27b-3p* was differentially expressed (higher in males) and had a sex-specific age trajectory (decreasing by age in males, increasing in females). A predicted regulatory target of hsa-miR-27b-3p is the transcription factor PPAR*γ*. PPAR*γ* has a multifaceted role in lung development, including the modulation of inflammatory and cell differentiation processes in the lung (Simon et al., 2006; Lecarpentier et al., 2019) and its negative feedback with the Wnt/*β*-catenin and TGFβ signaling pathways (Lecarpentier et al., 2019), two processes essential to branching morphogenesis (De Langhe and Reynolds, 2008; Saito et al., 2018). Disruption of PPAR*γ* in mice models alters lung volume and airway resistance (Simon et al., 2006). Consequently, the differences in the levels and age-trajectories of *let-7b-5p* and *miR-27b-3p* by sex observed here in human lung samples are one plausible mechanism underlying differences in lung histology and maturation rates between males and females during early human lung development.

Given the importance of pseudoglandular stage in airway development, it has long been hypothesized that insults to lung development during this period contribute to the developmental origins of chronic airway diseases (Stocks and Sonnappa, 2013). Impaired gene expression during this period likely alters susceptibility to chronic obstructive pulmonary disease (COPD) (Warburton et al., 2006) and asthma (Melén et al., 2011). These two diseases differ in etiology and pathophysiology by sex. Because sex-differing pseudoglandular miRNAs appear to regulate known airway development genes and pathways, our findings support the potential for sex-differing miRNA regulation very early in lung development to contribute to later sex differences in airway disease. Our group has previously shown that miRNA are biomarkers for abnormal lung function in asthmatic children in a sex-specific way (Kho et al., 2016b). In our current study, we note that our sex-differing miRNA results overlapped with miRNA markers derived from lung samples in COPD and asthma cases (Cañas et al., 2021), such as *hsa*-*miR-31-5p*, *hsa-miR-338-*3p, and again the *let-7* family with the same direction of effect. Importantly, lung development miRNAs that are differentially expressed by sex may be associated with the pathogenesis of several different pulmonary diseases by playing a key role in the inflammation that characterizes many chronic lung diseases such as cystic fibrosis, asthma, and emphysema (Sessa and Hata, 2013).

Our findings also have links to other diseases with known sex differences. For example, the higher levels of *miR-27b-3p* in pseudoglandular male lung is notable because PPAR*γ* agonists have been proposed for prevention and treatment of bronchopulmonary dysplasia (BPD) (Simon et al., 2006), a disease more common in males. We also detected sex-specific pseudoglandular trajectories (increase by age at greater rates in males) of *miR-29a-3p* and *mir-150*, two miRNAs with putative BPD-associations but elusive causal impacts on BPD severity in hypoxia-based experimental models (Nardiello and Morty, 2016). Interestingly, the respective predicted gene targets for these genes include collagen type 1 alpha 1 (*COL1A1*)/tropoelastin (*ELN*) and homeobox A5 (*HOXA5*)/C-Myc are known to have structural implications for early lung development (Schmidt et al., 2020).

To our knowledge, sex differences in miRNA expression in early in human lung development have not been previously profiled using high-throughput small RNA sequencing. Previous miRNA-sequencing studies in mice have focused on differential expression of miRNAs by sex during the later canalicular and saccular stages (Mujahid et al., 2013). Importantly, regulatory networks associated with murine miRNAs that are differentially expressed by sex during lung development have been shown to include androgen signaling (Mujahid et al., 2013). Furthermore, mouse models also show that miRNAs are modulated by androgens in developing lungs (Bouhaddioui et al., 2016). Importantly, many of the impacts of sex hormones established from mouse models in later lung development may still apply to earlier lung development. These observations include the finding that androgens alter fetal lung fibroblast maturation in murine lung *via* EGF and TGF*β* signaling events (Dammann et al., 2000). Additionally, androgens appear to block endogenous glucocorticoids, which are essential to normal lung development and are promoters of surfactant production (Provost et al., 2013). More broadly, androgens are thought to exert an inhibitory effect, while estrogens exert a stimulatory effect on lung development (Carey et al., 2007). Our results in early human lung development mirror the animal data in the later stages of gestation. Notably, pathway enrichment analysis of the predicted gene expression targets of our sex-specific miRNAs demonstrate enrichment in several sex-hormone related pathways, including both estrogen metabolism and androgen receptor signaling pathways.

We also demonstrate that the expression of several human fetal lung miRNAs was modified by age, demonstrating changes in gene expression across the pseudoglandular stage that varied by sex. These results also suggest a link to sex-steroid regulation of early lung development. The expression of *hsa-let-7b* increases across the pseudoglandular stage in males, while its expression does not change in females. *Hsa-let-7b*, a tumor-suppressor miRNA, has been previously shown to be important in both health and disease. In addition to its functions noted above, the let-7 family has also been shown to be an essential regulator of several endocrine systems. Transgenic mouse models have demonstrated that in combination with Lin28, the let-7 family of miRNAs are crucial to the timing of puberty and that overexpression of this dyad may result in the delayed onset of puberty (Zhu et al., 2010). While there are myriad mechanisms that regulate the expression of this system including dietary manipulation, it is well recognized that hormonal changes have been shown to have a significant impact (Sangiao-Alvarellos et al., 2015). Animal studies suggest that sex hormones may be partly responsible for the sex-specific differences seen in lung development. While these results suggest that sex-specific lung development miRNAs may influence sex hormone metabolism and signaling during the pseudoglandular stage of development, additional confirmatory studies are necessary to delineate the impact of lung development miRNAs on sex hormone levels.

Although these results provide a comprehensive evaluation of the sex-specific miRNA expression profile of early human lung development, there are several limitations to this study. Our samples were obtained from a fetal tissue biorepository with limited maternal and fetal information on each sample. Therefore, we could not comment on confounders such as maternal and fetal comorbidities. We attempted to address potential unmeasured confounders (i.e., unknown intrauterine exposures, maternal comorbidities, etc.) using the RUV method to adjust for unexplained miRNA expression heterogeneity, which includes possible unmeasured technical and biological confounders. We also limited our current study to investigation of the autosomal microRNAs. While there is a high density of miRNA on the X chromosome, the functional and statistical analyses of these loci remain complex and incomplete (Di Palo et al., 2020). We were unable to do validation of miRNA expression analyses due to the limitations in the quantity of RNA available from these extremely rare pseudoglandular samples. However, many of the predicted gene expression targets have been previously identified as demonstrated differential expression by sex in our earlier work suggesting the biologic plausibility of our current work (Kho et al., 2016a). In addition, our sex-specific miRNA expression analysis of lung development was limited to fetal lung tissue samples from the pseudoglandular stage of development. Therefore, we are not able to determine the changes in the sex-specific miRNA profiles that occur during the later stages of gestation, which may have implications for our understanding of subsequent disease risk. Additionally, the fetal sample age was estimated from days post-conception and not confirmed by histology, which could have introduced classification bias based on errors in estimation. Finally, although our results suggest that sex-specific miRNAs are important in lung development and may impact future disease risk, additional functional validation in animal models would be necessary to confirm the impact of altering these miRNAs on subsequent disease risk.

In conclusion, our study demonstrates sex-specific differences in miRNA expression between the male and female developing human lung and establishes their role in branching morphogenesis and airway development during the pseudoglandular time period. This study suggests that miRNAs may regulate the sex differences seen in lung development and that these differences in miRNA expression may be potential mechanisms to explain sex-specific differences in disease susceptibility to pulmonary disease later in life. Furthermore, our findings provide evidence that sex-specific miRNA expression profiles of lung development using human fetal lung tissues can be used to elucidate novel biological mechanisms that regulate the sex differences of developmental processes.

## Data Availability

The datasets presented in this study can be found in online repositories. The names of the repository/repositories and accession number(s) can be found below: https://www.ncbi.nlm.nih.gov/geo/, accession number: GSE200153.
