# Supplementary material for: Sex-Specific Differences in MicroRNA Expression During Human Fetal Lung Development
Source: Front Genet. 2022 Apr 11;13:762834. doi: 10.3389/fgene.2022.762834 (PMC9037032; doi:10.3389/fgene.2022.762834)
Supplement: Supplementary file 2 [file DataSheet2.docx]

**Supplementary Text 2. Comparison of Sex Effect Estimates by Model**

The effect estimates and identity of miRNAs identified to be differentially expressed with sex were compared between the two models (i.e., sex effect in model with no interaction, versus sex effect in the model allowing for an age-sex interactive effect).

As the former is presented throughout the text (main text **Table 2**, and **Supplementary Table S1**), in this supplementary file we evaluate whether presenting sex effect estimates from the other model would change any conclusions. The former is presented due to the decreased complexity of the regression model without interactive effects (see “Regression Modeling of miRNAs” in **Supplementary Text S1**).

The below venn diagram indicates the overlap in significantly differentially expressed miRNAs by sex in both models (q-value < 0.05). Ninety-two miRNAs were identified in both models. The model allowing for an interaction additionally detected hsa-miR-146b-3p (log_2_FC = 0.22, higher in males) and hsa-miR-381-3p (log_2_FC = -0.22, higher in females). One miRNA, hsa-miR-6721-5p, was detected in the main effect model exclusively.


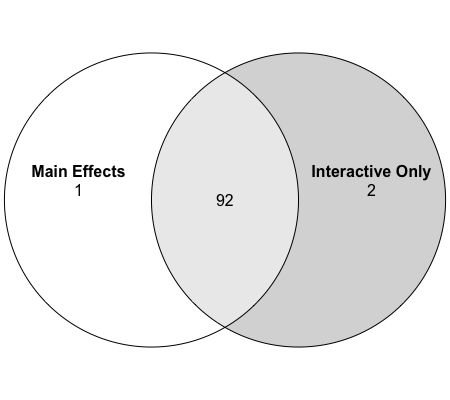

The magnitude and direction of the effect estimates (figure below, left) and p-values (figure below, right) are also very similar between the models.


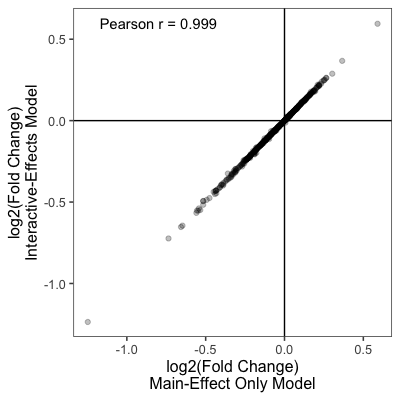

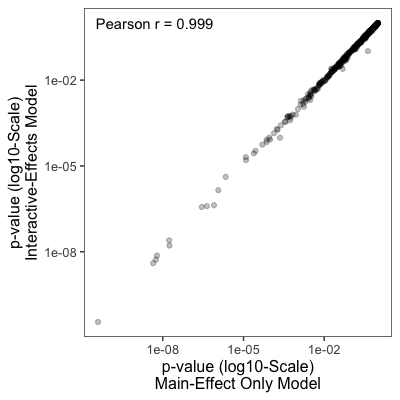


The conclusions regarding differential expression of miRNAs by sex are thus very similar regardless of the model used.
