# Supplementary material for: Sex-Specific Differences in MicroRNA Expression During Human Fetal Lung Development
Source: Front Genet. 2022 Apr 11;13:762834. doi: 10.3389/fgene.2022.762834 (PMC9037032; doi:10.3389/fgene.2022.762834)
Supplement: Supplementary file 5 [file DataSheet1.docx]

**Supplementary File 1. Detailed Methods**

Sample Acquisition and Metadata

Human fetal lung tissue samples were collected as part of a prenatal tissue retrieval program sponsored by the National Institute of National Child Health and Development, the University of Maryland Brain and Tissue Bank for Developmental Disorders (Baltimore, MD), and the Center for Birth Defects Research (University of Washington; Seattle, WA). The study was designated an institutional review board (IRB) exempt protocol by the University of Missouri-Kansas City Pediatric IRB, Partners Human Research Committee IRB, and the Colorado Multiple Institutional Review Board (COMIRB). Due to deidentification, limited metadata were available on each sample. We estimated gestational age using fetal foot length^1^ and inferred sample sex based expression of X- and Y-chromosome genes in paired microarray data^2^. Intrauterine cigarette smoke exposure (based on placental cotinine concentration)^3^ was measured using the Cotinine Direct ELISA kit (Calbiotech, Spring Valley, CA).

Small RNA Library Prep/Sequencing

We extracted total RNA from 30mg of homogenized prenatal lung tissue with the miRNeasy Mini Kit per manufacturer instructions (Qiagen; Valencia, CA, USA). Samples were block-randomized by age, sex, and smoke exposure status to four batches of miRNA library preparation (Small RNA Sequencing Kit v3 for Illumina Platforms; Bio Scientific) and sequencing (HiSeq2500; Illumina; San Diego, CA, USA). The randomization scheme was generated so that the proportion of samples with (i) male sex, (ii) intrauterine smoke exposure, and (iii) estimated ages above the median were approximately the same across all technical batches. Technical replicates (including prenatal lung samples, as well as a set of internal standard small RNA libraries) were included to examine the potential presence of technical effects in library preparation and during sequencing.

Bioinformatic Processing

Cutadapt^4^ v2.7 was used to trim low Q-score and adaptor sequence from each resulting .fastq file (cutadapt -u 4 -a NNNNTGGAATTCTCGGGTGCCAAGG -j 48 -q 20 -m 10 --trim-n -o). FastQC^5^ v0.11.8 was applied to each trimmed .fastq file to assess adaptor removal.

miR-MaGiC^6^ with reference to the *Homo sapiens* miRBase^7^ database v22.1 with collapsed functional groups (<https://github.com/KechrisLab/miR-MaGiC>; repo version 7147b7a) was used to process each trimmed .fastq into a count matrix of mature miRNA counts.

Subsequent steps were performed in using the R statistical package^8^, v4.0.2.

Sample-Level and miRNA-Level Quality Control

Samples were included in analyses if they had metadata available (on age, sex, and smoke exposure), estimated ages within the pseudoglandular histology time frame, > 4x10^5^ miRNA counts quantified by miR-MaGiC. Samples were aggregated within a sequencer run across lanes if they had high concordance across sequencing lanes.

miRNAs were included in the subsequent RUVSeq and DESeq2 modeling steps if they had non-zero counts in >25% of the samples passing quality control.

RUV Factors

RUVSeq^10^ v1.22.0 was used to control for unmeasured sources of expression heterogeneity. The variance stabilizing transformation (VST) of DESeq2^11^ v1.28.21 was applied to the data. Then, the VST-counts were regressed on age and age^2^ (polynominal contrasts applied to estimated days post-conception, using R function poly), sex (male/female), smoke exposure (< or ≥7.5 ng cotinine/g placenta), and batch (indicator for 1, 2, 3, or 4) in a linear regression model to obtain residuals. Finally, RUVr was applied to obtained 4 components. The number of components were selected by re-running RUVr for varying values and calculating the overall percent variance explained in the VST-counts using PERMANOVA, as implemented in vegan^12^, v2.5-7. The number associated with a plateau in percent variance explained was selected.

Exploratory data analyses (e.g., principal component analysis, visualization of a given miRNA’s counts across samples) were performed to identify putative outliers pre- and post-RUV solution.

Regression Modeling of miRNAs

Two models were then run using DESeq2, using the “local” method of dispersion estimation but default settings otherwise.

First, we identified miRNAs with varying average expression levels between male and female samples that persist across the entire pseudoglandular developmental stage. We modeled the count of each miRNA (outcome) by sample sex (explanatory variable of interest; indicator variable for male or female), adjusting for each of the following covariates: age, age^2^, smoke exposure, technical batch, and 4 RUVr components (each coded as described in the previous paragraph). This model is a “main effects” model. A statistically significant difference in mean miRNA levels by sex was defined by a negative binomial likelihood ratio test at a multiple testing corrected q-value^13^ < 0.05. The 539 miRNAs that were autosomal, passed the DESeq2 independent filtering criteria^9^, and converged in the regression modeling procedure were included in q-value multiple testing correction and reported.

Second, we screened for miRNAs with sex-specific “age-trajectories” to evaluate whether adding an age-by-sex interaction significantly improved model fit. A significant interaction (negative binominal likelihood ratio, q-value < 0.05) implies that the pattern of miRNA expression levels by age were distinct between male and female participants. For example, a miRNA may increase in male samples, yet decrease or remain the same in female samples. This model is a “interaction effects” model. We note that the RUVr solution with 4 factors was re-run for the interaction model, as inclusion of the age-sex interaction model results in different residuals. In our text, we discuss only linear age-by-sex interactions here for interpretability and because only two statistically significant quadratic (age^2^-by-sex) interactions were detected. As in the “main.effects” model without interaction, the miRNAs that were autosomal, passed the DESeq2 independent filtering criteria, and converged in the regression modeling procedure were included in q-value multiple testing correction and reported. Note that due to the greater complexity of the interactive model, fewer miRNAs passed independent filtering and model convergence; thus, only 441 miRNAs were tested.

Using this interaction effects model, we also tested whether the slope of the male miRNA age-trajectory significantly differed from zero using the Wald test (i.e., whether the regression coefficient of Age + Male*Age = 0) after correcting for multiple testing (q-value < 0.05). In addition, we tested whether the

Note that due to the coding with polynomial contrasts and quadratic effects, the values and effects sizes of age are difficult to interpret; however, we were interested primarily in identifying miRNA features that differ by sex for this work. Other work outside the scope of this manuscript is underway to characterize age patterns.

We also performed a sensitivity analysis in which we noticed extremely high similarities in the sex effect estimates: i.e., log_2_(fold change) values for sex were the same in the main effect model and interactive effect model.

Annotation of Regulatory Gene Targets

In our result tables, we provide some theoretical messenger gene targets theoretically regulated with each miRNA. These are based on the top ten predicted targets (if any) obtained from miRNAtap^14^, which aggregates an ensemble prediction of targets across five different prediction databases (v1.22.0, miRNAtap.db v0.99.10).

Pathway Analysis.

To interpret the functional impact of miRNAs differentially expressed by sex and with sex-specific age trajectories, we used miRNAtap^14^ to identify predicted gene targets regulated by each miRNA. In addition, we sorted the list of miRNAs tested by descending p-value and used preranked gene set enrichment analyses^15^ in miEAA (accessed November 2021)^16^ to conduct pathway analyses. The assignment of miRNAs to pathways was based on miRWalk v2.0 annotations^17^, as curated by the miEAA developers. Statistically significant enrichment in a pathway (Benjamini-Hochberg correction^18^; q-value < 0.10) indicates that miRNAs associated with the pathway appear at the top of the list (lower p-values) more frequently than would be expected by random chance. A more permissive multiple testing correction is used than in the primary analysis due to high redundancy between pathways. In pathway analysis results tables, only significantly enriched pathways are shown.

Obtaining miRNA-Residuals for Visualization

Figure 2 and Figure 3, we obtained residualized miRNA levels in order to show patterns after adjusting for covariates. These residuals were used for exclusively for visualization purposes, and not in subsequent statistical analyses. This adjustment was desired due to the influence of other covariates on miRNA expression: namely, age followed by technical effects (represented by the batch indicator and RUVr factors).

To visualize sex main effects (Figure 2), we thus regressed VST-counts on age, age^2^, smoke exposure, batch, and RUVr factors in a linear regression model. We did not include sex as a covariate; thus, the resulting residuals thus roughly will represent the variance in miRNA values attributed to all features besides sex.

Similarly, in order to visualize age-sex interactions (Figure 3), VST-counts were regressed on smoke exposure, batch, and RUVr factors.

Reproducibility

Code for statistical analyses and processed miRNA-sequencing data is available at github.com/chooliu/miRNASexDimorphismFetalLung.

References

1. Wong, H. S. A revisit of the fetal foot length and fetal measurements in early pregnancy sonography. *Int. J. Womens. Health* **Volume 9**, 199–204 (2017).

2. Kho, A. T. *et al.* Age, sexual dimorphism, and disease associations in the developing human fetal lung transcriptome. *Am. J. Respir. Cell Mol. Biol.* **54**, 814–821 (2016).

3. Vyhlidal, C. A. *et al.* Cotinine in Human Placenta Predicts Induction of Gene Expression in Fetal Tissues. *Drug Metab. Dispos.* **41**, 305–311 (2013).

4. Martin, M. Cutadapt removes adapter sequences from high-throughput sequencing reads. *EMBnet.journal* **17**, 10 (2011).

5. Andrews, S. FastQC: a quality control tool for high throughput sequence data. (2010). Available at: http://www.bioinformatics.babraham.ac.uk/projects/fastqc.

6. Russell, P. H. *et al.* MiR-MaGiC improves quantification accuracy for small RNA-seq. *BMC Res. Notes* **11**, 1–8 (2018).

7. Kozomara, A. & Griffiths-Jones, S. MiRBase: Annotating high confidence microRNAs using deep sequencing data. *Nucleic Acids Res.* **42**, 68–73 (2014).

8. R Core Team. R: A Language and Environment for Statistical Computing. (2015).

9. Bourgon, R., Gentleman, R. & Huber, W. Independent filtering increases detection power for high-throughput experiments. *Proc. Natl. Acad. Sci. U. S. A.* **107**, 9546–9551 (2010).

10. Risso, D., Ngai, J., Speed, T. P. & Dudoit, S. Normalization of RNA-seq data using factor analysis of control genes or samples. *Nat. Biotechnol.* **32**, 896–902 (2014).

11. Love, M. I., Huber, W. & Anders, S. Moderated estimation of fold change and dispersion for RNA-seq data with DESeq2. *Genome Biol.* **15**, 550 (2014).

12. Oksanen, J. *et al.* vegan: Community Ecology Package. (2016).

13. Storey, J. D. A direct approach to false discovery rates. *J. R. Stat. Soc. Ser. B (Statistical Methodol.* **64**, 479–498 (2002).

14. Pajak, M. & Simpson, T. I. miRNAtap: miRNAtap: microRNA Targets - Aggregated Predictions. (2020).

15. Subramanian, A. *et al.* Gene set enrichment analysis: A knowledge-based approach for interpreting genome-wide expression profiles. *Proc. Natl. Acad. Sci.* **102**, 15545–15550 (2005).

16. Kern, F. *et al.* miEAA 2.0: integrating multi-species microRNA enrichment analysis and workflow management systems. *Nucleic Acids Res.* **48**, W521–W528 (2020).

17. Dweep, H. & Gretz, N. miRWalk2.0: a comprehensive atlas of microRNA-target interactions. *Nat. Methods* **12**, 697–697 (2015).

18. Benjamini, Y. & Hochberg, Y. Controlling the false discovery rate: a practical and powerful approach to multiple testing. *Journal of the Royal Statistical Society B* **57**, 289–300 (1995).
